# Evaluation of whether there is residual polyp tissue after hysteroscopic morcellation at Cam and Sakura City Hospital: a retrospective cohort study

**DOI:** 10.1186/s12905-024-02978-4

**Published:** 2024-02-20

**Authors:** Mustafa Can Sivas, Karolin Ohanoglu Cetinel, Hilal Serap Arslan

**Affiliations:** 1 Department of Obstetrics and Gynecology, Republic of Türkiye Ministry of Health, Basaksehir Cam and Sakura City Hospital, Basaksehir Neighborhood, G-434 Street, No: 2L, Basaksehir, Istanbul, Türkiye; 2 Department of Pathology, Republic of Türkiye Ministry of Health, Basaksehir Cam and Sakura City Hospital, Istanbul, Türkiye

**Keywords:** Deficit, Endometrial sampling, Hysteroscopy, Morcellation, Polyp, Remnant

## Abstract

**Background:**

In polypectomy with mechanical hysteroscopic morcellators, the tissue removal procedure continues until no polyp tissue remains. The decision that the polypoid tissues were removed completely is made based on visual evaluation. In a situation where the polyp tissue was visually completely removed and no doubt that the polyp has been completely removed, short spindle-like tissue fragments on the polyp floor continue in most patients. There are no studies in the literature on whether visual evaluation provides adequate information at the cellular level in many patients in whom polypoid tissues have been determined to be completely removed. The aim of the present study was to analyze the pathological results of the curettage procedure, which was applied following the completion of polyp removal with operative hysteroscopy, and to evaluate whether there was residual polyp tissue in the short spindle-like tissue fragments that the mechanical hysteroscopic morcellator could not remove. The secondary aims of this study were to compare conventional loop resection hysteroscopy with hysteroscopic morcellation for the removal of endometrial polyps in terms of hemoglobin/hematocrit changes, polypectomy time and the amount of medium deficit.

**Methods:**

A total of 70 patients with a single pedunculate polypoid image of 1.5-2 cm, which was primarily visualized by office hysteroscopy, were included in the study. Patients who had undergone hysteroscopic polypectomy were divided into two groups according to the surgical device used: the morcellator group (*n* = 35, Group M) and the resectoscope group (*n* = 35, Group R). The histopathological results of hysteroscopic specimens and curettage materials of patients who had undergone curettage at the end of operative hysteroscopy were evaluated. In addition, the postoperative 24th hour Hb/HCT decrease amounts in percentage, the polypectomy time which was measured from the start of morcellation, and deficit differences were compared between groups.

**Results:**

In total, 7 patients in the morcellator group had residual polyp tissue detected in the full curettage material. The blood loss was lower in the morcellator group than in the resectoscope group (M, R; (-0.07 ± 0.08), (-0,11 ± 0.06), (*p* < 0.05), respectively). The deficit value of the morcellator group were higher (M, R; (500 ml), (300 ml), (*p* < 0.05), respectively). The polypectomy time was shorter in the morcellator group (M, R; mean (2.30 min), (4.6 min), (*p* < 0.05)).

**Conclusions:**

Even if the lesion is completely visibly removed during hysteroscopic morcellation, extra caution should be taken regarding the possibility of residual tissue. There is a need for new studies investigating the presence of residual polyp tissue.

## Background

Hysteroscopy is the gold standard for diagnostic and therapeutic tools used in the evaluation and treatment of conditions affecting the uterine cavity, such as abnormal uterine bleeding and infertility [1–3]. Conventional resectoscope hysteroscopy is the most common procedure performed to remove endometrial polyps using bipolar or monopolar electrical energy. Recent studies have shown that mechanical hysteroscopic morcellators that simultaneously cut and remove the targeted endometrial pathology are safe and effective alternatives to conventional hysteroscopy [4–6]. Advances in hysteroscopy instruments and ancillary equipment used in visualization have enabled especially mechanical hysteroscopic morcellators to be used in an office setting without the need for operating room conditions or general anesthesia [7, 8].

In operative hysteroscopy, the tissue removal procedure continues until no polyp tissue remains. The decision that polypoid tissues were removed completely is made by visual evaluation [7, 9]. To ensure that the root of the polyp is completely removed, some surgeons proceed slightly deeper into the myometrium [10]. In mechanical hysteroscopic morcellator, in a situation where the polyp tissue was visually completely removed and no doubt that the polyp has been completely removed, short spindle-like tissue fragments on the polyp floor continue in most patients, no matter how long the morcellation process was applied. In the literature, there is no study reporting whether short spindle-like tissue fragments belong to the endometrial tissue or the polyp.

In patients in whom polypoid tissues are determined to be completely removed, does visual evaluation provide sufficient information at the cellular level? Could the insignificantly small spindle structures that cannot be removed with a morcellator during morcellation, which are seen and perceived as the endometrial wall’s own tissue and considered unrelated to the polyp structure, be residual polyp tissue? We hypothesized that short spindle-like tissue fragments that the hysteroscopic morcellator cannot remove contain residual polyp tissue.

Therefore, the aim of the study was to analyze the pathological results of the curettage procedure, which was applied following the completion of polyp removal with operative hysteroscopy, and to evaluate whether there was residual polyp tissue in the short spindle-like tissue fragments that the mechanical hysteroscopic morcellator could not remove. Additionally, the aim of the study was to compare loop resection hysteroscopy with hysteroscopic morcellation in terms of hematocrit (HCT) / hemoglobin (Hb) changes, the amount of deficit and the duration of polyp removal.

## Methods

The study was designed and completed as a retrospective cohort study. This study was performed in line with the principles of the Declaration of Helsinki. Approval was granted by the Ethics Committee of the Basaksehir Cam and Sakura City Hospital local ethical committee (date: 28.09.2022, number: 2022.09.316/protocol no: 316).

### Inclusion and exclusion criteria

Patients who underwent hysteroscopic polypectomy between August 2020 and August 2022 were analyzed. Patients between the ages of 20 and 45 years were included in the study. Patient records were analyzed, and patients with a single pedunculate polyp 1.5-2 cm in size that was diagnosed during office hysteroscopy, and which was compatible with the operative hysteroscopy image, were included in the study. Patients in the menopausal or perimenopausal period, which could affect the study results due to atrophy or irregularity in the endometrial wall, or infertile patients with polyps were excluded from the study. All patients were in the proliferative phase of the menstrual cycle.

In light of the information obtained from the surgery notes, the patients who fully provided the following surgical steps in writing in the file records were included in the study. The inclusion and exclusion criteria were applied to 560 patients. Only 70 patients met all criteria. Statistical analysis was carried out for 70 patients, and the study was completed.

### Design of the study and surgical steps for the two groups

Patients who had undergone hysteroscopic polypectomy and subsequently underwent full curettage were divided into two groups, the morcellator group (Group M) and the resectoscope group (Group R), according to the surgical device used. Pathology results for both the hysteroscopy and curettage materials of all patients were obtained from the system records. Pathology results were analyzed to determine whether the hysteroscopic material was a polyp and whether there was residual polyp tissue in the curettage report. The deficit values stated in the surgical notes of the patients included in the study were compared between the two groups. The polypectomy time was calculated from the beginning to the end of the morcellation period. The blood Hb and HCT levels of all patients before and 24 h after surgery were analyzed from the system records, and whether there was a difference in the amount of blood loss between the two techniques was determined. The percentage decreases in Hb and HCT values were taken as a basis for determining the amount of bleeding.

In the morcellator (M) group, a hysteroscopy procedure (TruClear Elite Hysteroscope Mini, Medtronic Corp., Minneapolis, Minnesota, ABD) was started after cervical dilation of the Hegar bougie no.6. The morcellator system had a 6 mm diameter rigid hysteroscope and a 2.9 mm probe tip designed for polyp removal. Cavity distension was achieved by using a hysteroscopic pump (Hysterolux™ Fluid Management System, Medtronic Corp., Minneapolis, MN, USA), which provides constant pressure with 0.9% NaCl (saline) solution. The intrauterine cavity pressure was set to 100 mm/Hg. All morcellated tissue fragments were simultaneously suctioned and deposited in the filter system by means of the vacuum feature of the hysteroscope system. The vacuum pulling force was set to 150 mm/Hg.

In the resectoscope (R) group, an 8.5 mm diameter resectoscope (Rigid hysteroscope, Olympus Europa SE & Co., Wendenstraße, Hamburg, Germany) with a 24 Fr bipolar loop (bipolar resection electrode, Olympus) was introduced after cervical dilatation until the 9th bougie in the lithotomy position. Cavity distension was achieved by using a hysteroscopic pump (Hysteroflow-2, Olympus Europa SE & Co., Wendenstraße, Hamburg, Germany), which provides constant pressure with 0.9% NaCl (saline) solution.

The deficit volume was calculated by taking the difference between the amount of fluid used by the hysteroscopic pump while providing cavity distension and the fluid collected by the vacuum system or resectoscope drainage route [11]. After the operative hysteroscopy surgery was completed, all patients underwent routine full curettage to exclude possible malignancies or additional pathologies. No: 5 Karman cannula and a single-lock suction injector were used for the full curettage procedure. Polypoid tissue material and curettage material were sent for pathological examination in different containers as required by the routine practice of the hospital. The results of the hysteroscopy, which was performed by two surgeons (M.C.S., K.O.C.), and the pathology results of these operations that were reported by the same pathologist were collected from the records.

Within the framework of the routine surgical steps performed in our hospital, operations were performed under spinal anesthesia and in the standard lithotomy position. Full curettage was performed at the end after operative hysteroscopy operations as a routine practice. Additionally, after each operation, hysteroscopic polypectomy and endometrial curettage materials were carried in two separate containers, and the samples were subjected to pathological analysis separately. Polypectomy and endometrial curettage materials were evaluated by the same pathologist. Surgical materials were fixed in 10% formalin. Paraffin-embedded blocks and 5 μm sections were taken. Slides were stained with hematoxylin and eosin. Preparations were examined under a light microscope. The patient records were consistent with the routine surgical procedures (surgical steps and material handling/evaluation methods).

### Statistical analysis

When determining the sample size, calculations were performed in accordance with the protocol with gpower3.1 software (https://www.psychologie.hhu.de/arbeitsgruppen/allgemeine-psychologie-und-arbeitspsychologie/gpower), for a power value of 0.80, a margin of error of 0.05 and an effect size of 0.6. The Shapiro‒Wilk test was used to test whether the variables were normally distributed. Variables with a normal distribution are presented as the mean ± standard deviation, and an independent sample t test was used for comparisons between two independent groups. Variables that were not normally distributed are given median (minimum-maximum) values, and the Mann‒Whitney U test was used for comparisons between two independent groups. Categorical variables are presented as frequencies (n) and percentages (%), and Pearson’s chi-square test and Fisher’s exact test were used for comparisons. The statistical analysis was performed with the IBM SPSS Statistics 22.0 program (IBM Corp., Armonk, NY, USA). A p value of 0.05 was taken as the threshold for statistical significance.

## Results

A total of 70 patients were included in the study, 35 in the morcellator group and 35 in the resectoscope group. All of the hysteroscopy pathology results exhibited polyps. In addition to polypoid tissue, fibroid tissue was also reported in 2 patients in Group R and in 4 patients in Group M. According to the endometrial sampling results, in Group R, focal hyperplasia with atypia was observed along with residual polyp tissue in one patient, and focal hyperplasia with atypia without residual tissue was reported in one patient.

### Evaluation of descriptive statistics

No statistically significant differences were found in terms of age, gravidity, parity or type of delivery between the two groups (*p* > 0.05) (Tables 1 and 2). Moreover, there was no statistically significant difference in the hysteroscopy (H/S) pathology results between the two groups (*p* > 0.05) (Table 2).

**Table 1:** Age, gravidity, parity, HCT change (%), Hb change (%), deficit (ml) and polypectomy time (min) data

	Group	n	Median(Min-Max)/Mean ± SD	z/t	p
Age*	R	35	42(21–45)	-1.394	0.163
	M	35	40(22–45)		
Gravidity*	R	35	3(0–8)	-1.035	0.301
	M	35	2(0–9)		
Parity*	R	35	3(0–7)	-1.621	0.105
	M	35	2(0–9)		
HCT Change (%) **	R	35	-0.10 ± 0.06	-1.037	0.303
	M	35	-0.08 ± 0.091		
Hb Change (%) **	R	35	-0.11 ± 0.06	-2.017	0.048
	M	35	-0.07 ± 0.08		
Deficit*	R	35	300(100–2000)	-2.884	0.004
	M	35	500(100–2500)		
Polypectomy time*	R	35	4.6 ± 1.5	-6.373	0.001
	M	35	2.3 ± 0.2		

HCT: Hematocrit, Hb: Hemoglobin

*P* < 0.05, *Mann Whitney U Test, **Independent Sample t-Test

**Table 2:** Investigation of type of delivery, h/s pathology and residue status of the groups

			Group		p
			Resectoscope (*n* = 35)	Morcellator (*n* = 35)	
Type of Delivery *	No birth	n	4	3	0.653
		%	57.1%	42.9%	
	Vaginal Birth	n	15	18	
		%	45.5%	54.5%	
	Cesarean Section	n	6	8	
		%	42.9%	57.1%	
	Vaginal Birth + Cesarean Section	n	10	6	
		%	62.5%	37.5%	
H/S Pathology*	Polyp	n	33	31	0.673
		%	51.6%	48.4%	
	Polyp + Other	n	2	4	
		%	33.3%	66.7%	
Residue**	No	n	24	28	0.274
		%	46.20%	53.80%	
	Yes	n	11	7	
		%	61.10%	38.90%	

H/S: Hysteroscopy

*p* < 0.05, * Fisher’s exact test, ** Pearson’s chi-squared test

### Evaluation of the presence of residual tissue

In the morcellator technique, residual polyp tissues were detected in the endometrial samplings of 7 out of 35 patients. In the resectoscope technique, residual polyp tissues were detected in 11 of 35 patients. No statistically significant difference was found between the two groups (*p* > 0.05) (Table 2).

### Comparison of hematocrit/hemoglobin value changes, amount of deficit and polyp removal time between groups

In the morcellator system, the preoperative and postoperative Hb levels decreased from a median of 11.98 (8.7–14.6) g/dl to 11.18 (7.7–14.1) g/dl by 0.80. On the other hand, in the bipolar resectoscope system, Hb levels decreased from 11.68 (9.1–15.4) g/dl to 10.51 (7.9–12.8) g/dl by 1.17. A statistically significant difference was found in the comparison of the percentage decrease in preop-postop Hb values between the two groups (Group M (-0.07 ± 0.08) and Group R (-0.11 ± 0.06) (*p* < 0.05)) (Table 1). It was determined that there was less blood loss in patients who underwent surgery with the morcellator system.

In the morcellator system, the preoperative and postoperative HCT values decreased from a median of 37.41 (27.7–44.2) to 34.58 (26.8–44) by 2.83. Whereas in the bipolar resectoscope system, the median decreased from 35.85 (28.2–45.2) to 32.43 (25.9–38.7) by 3.42. No statistically significant difference was found in terms of the change in HCT percentage between the two groups (Group M (-0.08 ± 0.091), Group R (-0.10 ± 0.06) (*p* > 0.05)) (Table 1).

The median value of the deficit was 500 ml (100–2500) for the morcellation technique and 300 ml (100–2000) for the bipolar resectoscope method. The deficit values associated with the morcellation technique were higher than those associated with the resectoscope technique which was statistically significant (*p* < 0.05) (Table 1).

A statistically significant difference was found in the comparison of polyp removal time between the two techniques (Group M: 2.30 ± 0.2 min; Group R: 4.6 ± 1.5 min; *p* < 0.05).

### Comparison of all parameters according to the presence or absence of residual tissue

When patients with residual tissue and those without residue were compared in terms of age, gravidity, parity, type of delivery, H/S pathology, HCT percentage change, Hb percentage change and deficit values, ​​there were no statistically significant differences (*p* > 0.05) (Tables 3 and 4).

**Table 3:** Investigation of age, gravidity, parity, HCT change (%), Hb change (%) and deficit (ml) variables according to presence of residue

	Residue	n (%)	Median(Min-Max)/Mean ± SD	z/t	p
Age*	No	52 (74.3%)	40(25–45)	-0.169	0.886
	Yes	18 (25.7%)	40.5(21–45)		
Gravidity*	No	52 (74.3%)	2(0–7)	-0.736	0.461
	Yes	18 (25.7%)	2(0–9)		
Parity*	No	52 (74.3%)	2(0–7)	-0.92	0.357
	Yes	18 (25.7%)	2(0–9)		
HCT change (%) **	No	52 (74.3%)	-0.09 ± 0.07	-0.409	0.684
	Yes	18 (25.7%)	-0.09 ± 0.084		
Hb change (%) **	No	52 (74.3%)	-0.09 ± 0.07	0.106	0.916
	Yes	18 (25.7%)	-0.09 ± 0.07		
Deficit*	No	52 (74.3%)	500(100–2500)	-0.401	0.689
	Yes	18 (25.7%)	400(100–2000)		

Values are given in mean [95% confidence interval, standard deviation], median {range} or number (%).

HCT: Hematocrit, Hb: Hemoglobin

*p* < 0.05, *Mann Whitney U Test, ** Independent Sample t- Test

**Table 4:** Type of delivery and h/s pathology variables according to the presence of residue

			Residue		p
			No	Yes	
Type of Delivery	No Birth	n	5	2	0.775
		%	71.4%	28.6%	
	Vaginal Birth	n	25	8	
		%	75.8%	24.2%	
	Cesarean Section	n	9	5	
		%	64.3%	35.7%	
	Vaginal Birth + Cesarean Section	n	13	3	
		%	81.3%	18.8%	
H/S Pathology	Polyp	n	47	17	1.000
		%	73.4%	26.6%	
	Polyp + Fibroid	n	5	1	
		%	83.3%	16.7%	

H/S: Hysteroscopy

*p* < 0.05, Fisher’s exact test

## Discussion

In this study, it was demonstrated that residual polyp tissue fragments could remain in morcellator technique. Additionally, there was less blood loss in the morcellator system. Polyp removal time was shorter in the morcellator system, and the amount of deficit was detected more in the morcellator technique.

During resection with bipolar energy, many pieces of extracted tissue of various sizes are scattered in the cavity. Although visual control is achieved, polypoid tissue fragments which are released in the resectoscope technique may remain in the cavity. For this reason, false-positive residual polyps may have been detected in the curettage materials of Group R. In this respect, based on the literature review and analysis that we conducted during the study process, we think that a different sampling method is needed to more objectively evaluate the presence of residual tissue in the resectoscope group. In our study, we observed that the resectoscope technique was at least as successful as the morcellator technique in terms of the absence of residue remaining, and we found no significant difference between the two techniques. We have presented our data comparing the morcellator technique and the resectoscope technique in the Results section to provide insight for the reader. However, we find it more appropriate and prefer not to provide further comments due to the reasons we mentioned above. We think that there may be a significant difference in the resectoscope group as a result of evaluating the presence of residue in the resectoscope group with a different, more appropriate method.

On the other hand, the main point that our study investigated was whether there was any residual tissue when it was visually determined that the polypoid structure was completely removed in the morcellator technique. In the morcellator technique, there is a blade system at the morcellator probe tip that rotates back and forth. When the blade moves back, the window at the morcellator tip opens. The polyp tissue enters with the negative pressure effect of the vacuum system, and the window closes simultaneously as the blade moves forward and cuts the polyp. The polyp tissue piece that is released into a closed environment inside the morcellator tip is removed by a vacuum system. In addition, with the negative pressure effect provided by the vacuum, possible blood, debris or free tissue pieces in the uterine cavity are removed from the cavity as they are pulled through the window at the morcellator tip. As a result, the removed polypoid structures are simultaneously aspirated [5]. False positivity in the curettage material results of the morcellator group is therefore not expected. In these respects, our study results bring a valuable perspective to the literature in terms of drawing attention to the fact that residual tissue may remain in the morcellator technique.

There is no study in the literature providing histopathological data on whether residual tissue remains after polyp removal by operative hysteroscopy. One study is available aiming to investigate the recurrence of the polyp structure. In this study, the patients were followed for 4 years, the percentage of recurrent polyps was evaluated, and the use of the morcellation technique and bipolar resectoscope technique were compared. The recurrence rate was 9.8% in the bipolar system and 2.6% in the morcellator (TruClear) system [12]. In this study, it was examined whether new polyp tissue developed in the same patients who underwent surgery, within a 4-year period and whether secondary lesions were also considered polyps after surgical removal. In the same study, it was stated that whether the new polyp tissue, defined as recurrence, developed from the same location of the uterine cavity was checked and confirmed using the operating notes. However, even if the polyp originates from the same point in the uterine cavity, it is not possible to determine whether secondary polyp tissue developed from residual tissue that remained after the previous operation. It is always possible that the secondary polyp structure is a new cellular organization at the previous location in the cavity and may have affected the study. In addition, studies with longer follow-up periods are needed to determine whether these rates will change after more than 4 years. In this respect, our study tried to reveal the presence of residual tissue histopathologically following hysteroscopic polyp removal and evaluated the possibility of recurrence from a different perspective.

In terms of the decreases in blood Hb and HCT before and after the operation, we determined that there was less blood loss in the morcellator system. A statistically significant difference was detected in the comparison of the change in Hb percentages. Although there was no statistically significant difference in the percentage change in HCT, a larger percentage decrease was observed in the resectoscope group. We are of the opinion that statistical significance in the percentages of HCT change will be achieved in studies conducted with a larger number of patients.

Polyp removal time was shorter in the morcellator system. Previously published studies have shown that the morcellation technique reduces both the total operative time and the polyp removal time [5, 10, 13–15]. In our study, we obtained similar results on the basis of the polypectomy duration.

A larger deficit was measured in the morcellator technique. There is no consensus in the literature in terms of deficits. The applied intracavitary pressure, the vacuum pressure applied by the morcellator system, the surgeon’s experience and sleight of hand may have affected this parameter [5, 9, 16–18].

There was no perforation or any other complication in either technique. Although the low number of patients may not be sufficient to evaluate the frequency of perforation, perforation during the morcellation technique was found to be less common in the literature [9, 17].

This study is the first in the literature to evaluate whether residual polyp tissue remains after hysteroscopic morcellation. Similarly, this is the first study in the literature comparing the two techniques in terms of the degree of decrease in preoperative-postoperative hemoglobin and hematocrit values. This study contributes to the literature by comparing the two techniques in terms of polypectomy duration and deficit volume.

### Limitations of the study

The first limitation of this study is that it is necessary to evaluate the presence of residue in the resectoscope group with a different, more appropriate method. Therefore, this study does not have sufficient standardization to compare the success of the two techniques. For this reason, no detailed comparative comments were made between the two techniques. The second limitation is that the presence of residual polyp tissue was investigated in the pathology results of curettage procedure which sampling the entire cavity. Although it was visually confirmed that there was no secondary polyp tissue in the cavity, using a technique that samples only the area where short spindle-like tissue fragments are present will make the study more powerful. Another limitation is the small patient population. More patients are needed to evaluate the frequency of perforation complications and the change in HCT percentage between the two groups.

## Conclusion

Most surgeons visually determine that the polyp tissue is completely removed and terminate the operation during operative hysteroscopy. Even if the lesion is completely visibly removed during hysteroscopic morcellation, extra caution should be taken regarding the possibility of residual tissue. There is a need for new studies investigating the presence of residual polyp tissue.

## Data Availability

The datasets generated and/or analysed during the current study are not publicly available due to local ethical and legal requirements but are available from the corresponding author on reasonable request.

## Abbreviations

HCT: Hematocrit
Hb: Hemoglobin
M: Morcellator
R: Resectoscope
H/S: Hysteroscopy
